# A method for studying decision-making by guideline development groups

**DOI:** 10.1186/1748-5908-4-48

**Published:** 2009-08-05

**Authors:** Benjamin Gardner, Rosemary Davidson, John McAteer, Susan Michie, the "Evidence into Recommendations" study group

**Affiliations:** 1 Centre for Outcomes Research and Effectiveness, Department of Clinical, Educational and Health Psychology, University College London, 1-19 Torrington Place, London, WC1E 7HB, UK

## Abstract

**Background:**

Multidisciplinary guideline development groups (GDGs) have considerable influence on UK healthcare policy and practice, but previous research suggests that research evidence is a variable influence on GDG recommendations. The Evidence into Recommendations (EiR) study has been set up to document social-psychological influences on GDG decision-making. In this paper we aim to evaluate the relevance of existing qualitative methodologies to the EiR study, and to develop a method best-suited to capturing influences on GDG decision-making.

**Methods:**

A research team comprised of three postdoctoral research fellows and a multidisciplinary steering group assessed the utility of extant qualitative methodologies for coding verbatim GDG meeting transcripts and semi-structured interviews with GDG members. A unique configuration of techniques was developed to permit data reduction and analysis.

**Results:**

Our method incorporates techniques from thematic analysis, grounded theory analysis, content analysis, and framework analysis. Thematic analysis of individual interviews conducted with group members at the start and end of the GDG process defines discrete problem areas to guide data extraction from GDG meeting transcripts. Data excerpts are coded both inductively and deductively, using concepts taken from theories of decision-making, social influence and group processes. These codes inform a framework analysis to describe and explain incidents within GDG meetings. We illustrate the application of the method by discussing some preliminary findings of a study of a National Institute for Health and Clinical Excellence (NICE) acute physical health GDG.

**Conclusion:**

This method is currently being applied to study the meetings of three of NICE GDGs. These cover topics in acute physical health, mental health and public health, and comprise a total of 45 full-day meetings. The method offers potential for application to other health care and decision-making groups.

## Background

Evidence-based clinical practice is premised on developing healthcare guidelines informed by systematic reviews of research evidence. In the UK, the National Institute for Health and Clinical Excellence (NICE) commissions Guideline Development Groups (GDGs) tasked with formulating recommendations for clinical and public health practice on the basis of evidence from scientific research and other sources. GDGs comprise academic, professional and lay representatives from relevant disciplines and practices. Clinical GDGs typically meet around fifteen times over an eighteen-month period to consider research evidence and recommendations. GDG discussions are informed by verbal and written presentation of research evidence by systematic reviewers and health economists, comments on draft recommendations by stakeholders and sometimes contextual evidence from co-opted experts.

Health professionals and organisations in England and Wales are expected to use NICE guidelines to set standards for healthcare policy and decision-making [1]. Despite the potential influence of clinical guidelines on healthcare practice, little is known about the processes by which GDGs translate evidence into recommendations. These processes may not be straightforward. A study of 15 clinical guidelines on management of Type 2 diabetes from 13 countries found that only 18% of citations were shared with any other guideline, and only 1% appeared in six or more guidelines [2]. Similarly, a study of two independent expert panels formulating appropriateness criteria for investigation of patients with angina found that, given the same evidence summary and using a formal consensus process, the two groups showed only moderate agreement in their recommendations (Hemingway et al, personal communication). Thus, research evidence may not be the most powerful influence on the content of recommendations. Guideline development processes are thus open to influences which may result in recommendations being formed which are not based on the best available evidence. This in turn is likely to impact on guideline quality, implementation, and effectiveness [3,4]. Ensuring that guidelines are based on the best available evidence will depend on identifying these influences on GDG decisions.

The few studies available in this area have highlighted various social psychological influences on GDG decisions: conceptualisations of recommendations and evidence, and evaluation of different types of evidence [5]; beliefs and values [6,7]; professional status, interests, and opinions [8,9]; and the knowledge and experience of the group members in evidence evaluation and synthesis [6]. Additionally, small group processes (e.g. conformity, compliance) are likely to impact on guideline development [3]. These influences may compromise the quality of guideline recommendations. Dominance of some group members at the expense of others, for example, may weight GDG decisions in favour of one disciplinary perspective, which may adversely affect the acceptance and implementability of subsequent recommendations [3]. Similarly, shared conceptualisations of the guideline process as a consensus building rather than critical appraisal process may prevent members from considering all relevant information [3,10].

Improving GDG decision-making necessitates identification of influences on GDG decisions, as a basis for intervention. The 'Evidence into Recommendations' (EiR) study has been set up to investigate social-psychological influences on guideline formation, paying particular attention to who has most influence on group decisions, the strategies used in formulating recommendations, beliefs that may explain these strategies, and consequences for the quality of GDG process and outcome [11]. Social psychological theories of group processes are available which offer integrated summaries of potential influences on group decisions. For example, the 'groupthink' model suggests that group cohesion and the prioritisation of unanimity rather than quality can result in decisions of suboptimal quality [10]; social impact theory suggests that social status, power and credibility can impact on group members' willingness to favour decision options [12]; and a recent ecological model suggests that decision quality is a function of the extent to which information and preferences are shared among members [13]. These theories specify explicit pathways by which social variables impact on group decision-making. Applying theory to the study of group decision-making allows for a body of scientific knowledge on the functioning of small groups and communication between members to be drawn upon and new evidence accumulated within standardised and systematic frameworks. Moreover, in specifying determinants of group decisions, theory can offer potential targets for interventions aimed at improving decision quality. Yet theory is rarely used in investigations of GDGs [3]. The EiR study thus aims to provide an account of the social dynamics of decision-making based on theory and evidence, and to identify areas of good (and bad) practice. In so doing, it is intended that findings from the EiR study will inform guidance designed to raise awareness among GDG members of social processes that may impact on GDG decisions, so reducing problems which may prevent best-quality decisions being made (e.g. marginalisation or dominance based on professional status; [8]). This paper outlines the method which will be used in pursuit of the research objectives of the EiR study.

Capturing the guideline development process and factors that impact upon it presents a considerable methodological challenge. An experimental approach in which the presence of hypothesised influences is systematically varied [e.g. [14]], may reveal important insights but cannot be applied to naturalistic settings. Observation-based methods are therefore required. A study of four meetings of one GDG, drawing upon theories of small group processes and using 'interaction process analysis' to code group members' utterances, demonstrated both the task orientation of group discussion, and the influence of professional role and status on contributions to these discussions [8]. Interaction process analysis, which involves assigning one of twelve codes to each group member utterance (e.g. positive feedback given, direction offered, question asked; [15]) does not however sufficiently engage with the subject matter of group discussion. Consequently, it is difficult to distinguish between key exchanges and interactions that are not directly related to the main decisions. Qualitative methods may be better suited to identifying and understanding the content of interactions most central to GDG recommendations.

A qualitative analysis of verbatim transcripts of meetings of two GDGs used 'grounded theory' techniques to develop a coding structure and explore discursive domains around which discussions were organised [5]. Initial 'open' coding of data from verbatim meeting transcripts identified analytical categories, and data pertaining to these categories was subsequently extracted from across the dataset. Four different criteria for evaluating research evidence were identified (technical robustness, usability, acceptability, and methodological adequacy). This method of analysis demonstrates the potential for inductive techniques to identify and explore recurrent themes in GDGs, and to inform data reduction and extraction procedures. However, this method was limited for two reasons. Firstly, analysis was dependent exclusively upon researchers' interpretations of significant occurrences or discourses within the group, but researchers may overlook events deemed important by group members. Secondly, exclusively inductive analyses may neglect important insights from the theoretical or empirical literature. Theory and evidence relating to intra-group processes is likely to provide a useful basis for categorising influences on GDG discussions and organising analysis [3,8].

Previous observational studies have demonstrated the usefulness of qualitative methodologies for providing insight into GDG processes, but have typically focused on GDG meeting data alone, and have employed one methodological approach in isolation. Systematically gathered data from both GDG meetings and members' reflections on GDG proceedings may be required to permit a comprehensive analysis of social-psychological influences on GDGs. Additionally, reliance upon any one particular analytic method in isolation may limit the extent to which analysis can identify and engage with significant events within the GDG process, and draw upon theoretical and empirical insights into intra-group processes to understand these events. A pluralistic methodological approach is likely to be better suited to addressing the EiR study objectives.

This paper has two aims: firstly, to evaluate the usefulness of existing qualitative methodologies for the EiR study, and secondly, to develop for use in the EiR study a detailed, pluralistic method which integrates the most relevant techniques from existing methodologies. Hence, this paper outlines the development of a systematic method, which comprises both inductive and theory-informed coding techniques drawn from extant methodologies, to enable qualitative analysis of social psychological influences on GDG decision-making.

## Methods

### Design and data

The EiR study uses a longitudinal observational design to study three GDGs (one each from acute physical health, mental health, and public health). Our method utilises data from several sources: 1) verbatim transcripts of GDG meetings; 2) semi-structured interviews conducted at the start and end of the GDG process with a purposive sample of GDG members, selected to represent different constituencies within the group (e.g. academics, patient representatives, chair); and 3) stakeholder comments on GDG recommendations.

The EiR study has received ethical approval from the Research Ethics Committee of the UCL Psychology Department (ref: 0819/001). All GDG members provide written consent prior to data collection.

### Contributors

The research team comprises three Research Fellows (RD, BG, JM), and an eight-person multidisciplinary steering group, comprising senior academics (SM, GF, SP, RR, PD), and NICE staff from the public health guidance (SE) and clinical guidelines programmes (FC, PA). The method was developed over ten meetings, conducted over an eighteen-month period. All research team members have experience of sitting on GDGs, either as members (PA, FC, PD, SE, GF, SM, SP, RR) or observers (BG, RD, JM). Academic research team members' disciplinary background spans social, health and clinical psychology (BG, JM, SM, SP), sociology (RD), medicine and health services research (GF, PD, RR).

### Procedure

#### Scoping literature review and applicability task

The principal Research Fellow (RD) conducted a scoping literature review to identify extant qualitative analysis methodologies. Summaries of these were presented to the research team, which completed a task assessing the applicability of each method and its component techniques against two criteria: 1) usefulness for potentially permitting data reduction and 2) usefulness for incorporating theory- and evidence-specified relationships into our analysis. The relevance of each method for this task was subsequently discussed by the group. A configuration of techniques judged useful for the EiR study was agreed among the research team and piloted using four interview transcripts and a meeting transcript.

## Results

### Usefulness of existing methodologies

Methods identified by our review were: discourse analysis [16]; grounded theory [17,18]; content analysis [19]; conversation analysis [20]; thematic analysis [21]; interpretative phenomenological analysis [22]; and framework analysis [23]. Coding techniques derived from four methodologies (thematic analysis, grounded theory, content analysis, framework analysis) were judged to be most useful for our purposes (see Table S1, Additional file 1).

### A method for studying guideline development groups

**Figure 1:**
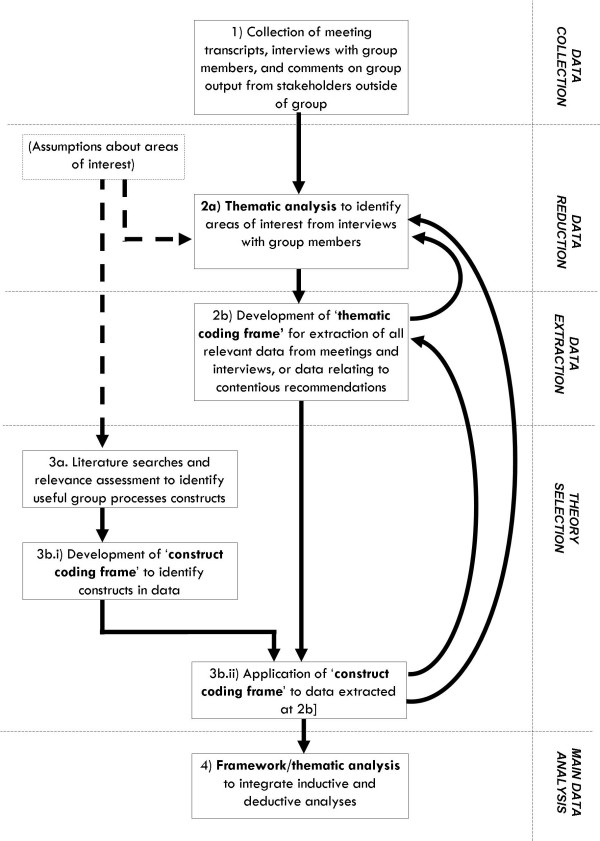
**Diagram of method**.

The method consists of four stages: 1) data collection, 2) data reduction, 3) selection and application of theory, and 4) main data analysis (see Figure 1). All coding procedures are piloted by multiple independent researchers and results discussed to ensure reliable and consistent coding. Procedures need not necessarily be performed sequentially; insights from later stages of analysis may inform refinement of concepts identified at earlier stages.

### Data collection

The aim of this stage is to gather evidence relating to, and which sufficiently encapsulates, GDG proceedings and members' experiences of these.

Audio recordings of each group meeting are transcribed verbatim. Semi-structured interviews are conducted at the start and end of the GDG process with a sample of GDG members, selected to represent the different constituencies of the group (e.g. service providers, academics, service users, GDG chair). Interview topics include: expectations and experiences of the group process; perceived task demands; roles of self and others within the group; significant incidents of disagreement and agreement; and representativeness of viewpoints within the group. (The interview schedule is available from the authors.) Stakeholders' reactions to GDG recommendations at the end of the GDG process are also retained for analysis.

Our dataset thus comprises transcripts of 45 meetings (15 meetings per GDG) and 70 interviews (2 interviews × 10–12 members × 3 GDGs), and 3 sets of stakeholder comments (one set per GDG).

### Data reduction

The second stage of the method is designed to condense the dataset, while retaining key features of interest to our research questions, prior to the main analysis phase. This involves systematically reducing data via application of a coding frame, developed from a broad thematic analysis of a subset of data, to extract data excerpts warranting further analysis.

#### Identifying areas of interest

One key assumption made by the research team, on the basis of first-hand experiences of GDGs and knowledge of social psychology theory (e.g. 11, 13), is that influences on within-group decisions will be best revealed by focusing on instances whereby members voice agreement or disagreement, or there is conflict or harmony when discussing the content of a potential decision. At the first stage of data reduction, these instances are identified and retained for further analysis. Free coding of interview transcripts is used to identify a) events or instances characterised by intragroup tension, conflict, disagreement, or dispute, b) instances of agreement and concordance, or c) any other incident of apparent significance to intragroup relations or GDG decisions. Thematic analysis [21] is used to assign provisional thematic labels to these events according to the focus of the dispute or agreement. Resultant themes represent areas of interest warranting further analysis.

#### Development and application of a 'thematic coding frame'

Constant comparison [17] is used to identify properties common to each theme. This informs the development of a 'thematic coding frame' comprising indicators of each identified theme. This allows identification of significant events from elsewhere in the dataset. The coding frame is applied to GDG meeting transcripts to extract passages of discussion in which instances of each theme are apparent. Start and end points of these passages are denoted by turning points in conversation or argumentation, or announcement of a decision.

Events that appear indicative of themes not previously identified, or that appear to pertain to identified themes but are insufficiently captured by the thematic coding frame, are noted. These are used to modify the thematic coding frame, allowing new themes and/or indicators of existing identified themes to be added. In this way, the coding frame is continually refined in response to the data until a definitive thematic coding frame is established.

Data extraction using the thematic coding frame proceeds in two ways. Firstly, data relating to each theme is extracted from each meeting transcript to identify and track the development of the theme through the course of the GDG. In this way, themes of apparent importance to the process of decision-making are the focus of analysis, and the association between each theme and key outcomes (i.e. GDG decisions and recommendations) can be explored.

A second strand of analysis is driven by a focus on one or more contentious GDG recommendations, and only data relating to the processes antecedent to the formation of these recommendations extracted. In this way, decision-making outcomes guide the analysis, and the tensions, conflicts, and agreements that have produced these outcomes can be documented. Conducting the outcome-driven analysis requires content analysis of stakeholder comments on GDG recommendations to identify phrases indicating stakeholder disagreement with GDG decisions (e.g. 'disagree', 'not acceptable', 'reservations', 'misleading', 'no evidence'). Sections of group discussions and interview data relating to the contentious recommendation(s) are identified, and the thematic coding frame is thus applied to further reduce these data.

Both procedures reduce the dataset to a series of pertinent discussions likely to be significant for intragroup relations and group decision-making.

### Selection and application of theory

The third stage of our method aims to identify theories and evidence to use as bases for coding data excerpts identified at stage 2. Relevant intragroup interaction theories and evidence, identified via systematic search procedures, inform a second coding structure which is applied to identify key concepts in our themed data excerpts.

#### Literature searches and relevance assessment

A search of social psychology and group decision-making textbooks is conducted to ascertain theories and evidence likely to be applicable to understanding group processes. Three criteria are applied to assess the relevance of these insights for our analysis: a) constructs explain a process of relevance to naturalistic group interaction (as assessed via exploration of our areas of interest; see section 2a above); b) empirical evidence from at least two independent studies supports the use of the construct to explain this process; and c) the construct can be operationalised for application to written accounts of naturalistic group interaction and/or individual interviews.

Notwithstanding our assumptions regarding incidents of interest within the dataset (see Section 2a, above), stage 3a of the method is not informed by outputs from stages 2a and 2b: the relevance of available theories and evidence is evaluated at stage 3a prior to application of these to the data excerpts identified at stage 2b. In this way, data extraction at stages 2a and 2b does not constrain or otherwise influence judgements about the utility of extant theories or evidence for our analysis.

#### Development and application of a 'construct coding frame'

At this sub-stage, theories and evidence deemed relevant at stage 3a are used to code the data excerpts extracted at stages 2a and 2b. Indicators of constructs meeting the three criteria (outlined at stage 3a) are developed both deductively and inductively to enable identification of each construct in the data excerpts. Operationalisations of each construct draw upon reliable or theoretically valid measures used in previous research studies, empirical evidence regarding proxy indicators of the construct, and/or conceptual definitions of the construct. Additionally, a small and randomly selected portion of data excerpts is inductively coded to identify apparent instances of each construct not sufficiently captured by our operationalisations. This informs the development of a 'construct coding frame', to facilitate systematic and reliable identification of incidents pertinent to each construct within the data excerpts. Pilot application of the construct coding frame assesses its utility, and any problems inform subsequent refinements to the coding frame.

The construct coding frame is applied to sections of transcript retained at step 2. Application of the definitive 'construct coding frame' to the data excerpts allows us to understand the themed data extracts using concepts derived from group processes theory and evidence.

### Main data analysis

The final stage of the method aims to bring together the themed data excerpts from stage 2, as coded for their theoretical content at stage 3, so as to develop and structure explanatory accounts of each theme.

This stage draws upon thematic and framework analysis procedures. Framework analysis is a qualitative method which fuses deductive and inductive enquiry by permitting analysis to be guided by preconceptions regarding relationships between constructs, and their antecedents and consequences, but also facilitates re-specification of these relationships and the identification of additional links and pertinent concepts emerging from the data [23].

A framework is constructed which comprises each of the previously identified themes and concepts subsumed within these themes at step 2, and the theory-based constructs found to associate with each of these themes at step 3. This framework is applied to data previously extracted from meeting and interview transcripts.

'Thematic matrices' are constructed to visually display data relating to each of the concepts grouped together under an overarching theme, and to enable emergence of relationships between these concepts and GDG decisions. Theory-based constructs, as coded within the data at stage 3, are drawn upon where these enrich understanding of or otherwise characterise these relationships. The analysis thus explores commonalities, causes and consequences of inductively identified concepts, using where possible constructs deductively derived from the theoretical and empirical literature as potential explanatory mechanisms for these links.

The framework is responsive to insights emerging from the data, and where, for example, the concepts and themes imposed by the framework appear to be mislabelled or new concepts emerge, the framework is refined. Several iterations are undergone to develop a definitive framework which documents patterns of association between concepts underpinning key themes within the GDG decision-making process.

### An illustration

We are currently applying our method to the study of an acute physical health GDG. The illustration below is designed to show how our method has been applied to this GDG, and the type of insights that may emerge from its application, thus testifying to the utility of the method. Analysis is ongoing, and so results are tentative and not intended to reflect the output of a comprehensive application of our method.

#### Stage 1: Data collection

Data relating to the acute physical health GDG comprises 15 meeting transcripts, 24 group member interviews, and one set of stakeholder comments.

#### Stages 2a and 2b: Data reduction

Three overarching themes emerged at stage 2a. One theme, relating to the nature and applicability of 'evidence', subsumes sub-themes relating to: conceptualisations of 'evidence' and its role in recommendations; decision-making in the absence of high-quality evidence; clinical judgement versus research evidence; and references to own professional experiences. A second theme refers to diversity and hierarchy, and incorporates sub-themes of: lay and professional perspectives; challenges of multi-disciplinarity; and minority voices. A third theme addresses contextualising recommendations, and encompasses references to other guidelines, resource implications, and framing recommendations around available evidence and/or clinical need. Operationalisation of these themes into a coding frame (step 2b) facilitated extraction of excerpts relating to these themes.

#### Stages 3a and 3b: Selection and application of theory

One of the theories identified and adjudged relevant at stage 3a relates to self-categorisation and social identity [24]. The concept of 'social identity' (i.e. a person's self-concept as defined by her or his group membership [s]) has been shown to be associated with favourability for one's own social group(s), and denigration of other groups [24]. Following identification of social identity theory as relevant, data excerpts identified at stages 2a and 2b were coded using concepts from this theory.

#### Stage 4: Main data analysis

One excerpt for which the application of identity-related concepts has been useful details an exchange in the early stages of the GDG process. The group is discussing the setting of clinical questions to be addressed by the GDG. A clinician pre-empts the discussion by asserting a recommendation that he feels should be made, despite the group not having assessed the research evidence at this point. The clinician advises systematic reviewers to seek evidence to support this recommendation. In doing so, the clinician suggests that professional opinion be prioritised over research evidence in shaping the clinical question and determining the scope of literature searches and evidence evaluation. This operates to discount the views of non-clinician group members who do not share clinicians' professional expertise and so conversely elevate the importance and status of clinicians within the group.

## Discussion

The 'Evidence into Recommendations' (EiR) study aims to develop an understanding of social psychological influences on decision-making among guideline development groups (GDGs), so as to inform interventions to reduce the likelihood of suboptimal quality GDG decisions being made. Identifying and capturing the development of these processes over multiple meetings and their influence on the many decisions of the GDG process is a challenging task and is likely to require a pluralistic methodological approach. We have evaluated existing qualitative methodologies and techniques for their usefulness for the EiR study, and developed a method for collecting data relating to GDG decision-making and for understanding and making inferences from these complex data. Our method incorporates recommendations for sources of data (verbatim meeting transcripts, interviews with group members, feedback on the acceptability of group recommendations from stakeholders external to the group), procedures for extracting pertinent data from these sources, techniques for applying theoretical and empirical insights into group processes to code these extracts, and a structure for integrating these stages into an overarching qualitative analysis. Analysis is undertaken using a unique configuration of techniques drawn from various extant qualitative methodologies (thematic analysis [21], grounded theory [17], framework analysis [23]). Consequently, our method illustrates the potential both for innovation by synthesis in qualitative analysis, and for qualitative methods to be flexible and adaptable to research demands.

Stages of our method are not intended to be necessarily sequential. Insights which emerge from, for example, the group processes literature may inform refinement of the themes identified in the preliminary thematic analysis. Hence, our method is flexible and responsive to developments in the analysis procedure.

Our method integrates inductive and deductive methods to produce a qualitative analysis that is attentive to concepts emerging from the data but also allows for these to be interpreted in light of extant theory and research evidence. Additionally, in developing data extraction and coding structures on the basis of insights from interviews with GDG members, our analysis is guided by members' reflections on important incidents within the group. In this way, we minimise potential problems inherent in relying upon researchers' interpretations of significant events.

Our method is designed to capture and describe the processes which influence group decision-making within GDGs. We note that our method is itself the output of a group-based decisional process, developed on the basis of discussions among a multidisciplinary research team. While it is not our objective to document the processes involved in the production of our methodology, it is noteworthy that the method with which we will explore group interaction in the EiR study may have been shaped by the very group processes under examination. We do not however view this as problematic, because practical application of our method may inform subsequent iterative refinements to the method.

The method we have presented centres on the collection and analysis of textual data to allow us to address the research questions of the EiR study [10]. More fine-grained analyses may be possible where non-verbal data is available, for example via transcription of audio recordings using systems which map aspects of speech delivery (e.g. tone) and temporal relationships within verbal interactions [25], and/or the collection and analysis of video recordings of group discussions. Analysis of non-verbal communication is beyond the scope of the EiR study. Further work might develop our analytic framework so as to incorporate analyses of non-verbal data into a multi-level qualitative analysis of GDG decisions.

We are currently applying our method to study GDGs in acute medicine, mental health and public health, and unforeseen problems may arise in application which require refinement of the method. Initial findings suggest however that the method we have presented allows for a potentially more comprehensive analysis of GDG decisions than has been achieved previously. Additionally, the method is likely to be useful for studying the formation of decisions by other healthcare groups.

## Competing interests

The authors declare that they have no competing interests.

## Authors' contributions

SM conceived and developed the study, and leads its implementation. RD, the principal Research Fellow, coordinates the ongoing study, and collected data. RD, BG, JM and SM developed and piloted the method. BG drafted the manuscript, which was refined in light of comments from JM, SM and RD, and two peer reviewers. The 'Evidence into Recommendations' study group contributed to assessment of extant analysis techniques. All authors read and approved the final manuscript.

## Supplementary Material

Additional file 1 **Table S1**. Extant qualitative methodologies considered for inclusion in our method. Click here for file
