# Supplementary material for: A method for studying decision-making by guideline development groups
Source: Implement Sci. 2009 Aug 5;4:48. doi: 10.1186/1748-5908-4-48 (PMC2731071; doi:10.1186/1748-5908-4-48)
Supplement: Additional file 1 — Table S1. Extant qualitative methodologies considered for inclusion in our method. [file 1748-5908-4-48-S1.doc]

Additional file 1 – Table S1. Extant qualitative methodologies considered for inclusion in our method

| *Methodology* | *Summary* | *Epistemological orientation* | *Research team comments and judgements on usefulness for EiR study* | | |
| --- | --- | --- | --- | --- | --- |
|  |  |  | *General* | *Usefulness for data reduction* | *Usefulness for imposing theory/evidence-based relationships on data* |
| Discourse analysis [16] | Argues that language is active and aims to reveal how language is used to construct a particular version of reality. | Constructionist  (i.e. assumes that there is no objective ‘reality’, only that created and negotiated socially. Contrasts with ‘realism’, which assumes an objective reality that can be accessed through scientific enquiry) | Constructionist epistemology conflicts with the realist assumptions inherent in the EiR study research questions. | Not useful | Not useful  (social psychological theories of group processes are realist and so incompatible with DA) |
| Grounded theory [17, 18] | The grounded theory method provides guidelines on how to extract categories from the data, and to establish links between these. Analysis must be inductive – i.e. argues that categories inherent in the data should be uncovered by analyst, and that the analyst should not impose preconceived theoretical expectations on the data.  Data are coded using well-defined procedures and analytic tools, including the constant comparison method, which involves comparing instances of similarly labelled concepts to identify commonalities in components of each of the emergent categories. | Can be realist or constructionist. | Constant comparison method likely to be helpful as a generic tool. | Useful  (constant comparison method may aid conceptualisation / development of emergent themes) | Not useful  (emphasis on inductive analysis inappropriate for linking theory and evidence-driven analysis) |
| Content analysis [19] | Involves coding data into discrete categories or themes, developing these themes inductively from the data, and, usually quantifying themed material.  Initial analysis stages are similar to thematic analysis, but differ in requiring analysis be inductive. | Realist |  | Useful  (data could be selected for analysis according to whether they fit emergent themes) | Not useful  (emphasis on inductive analysis inappropriate for linking theory and evidence-driven analysis) |
| Conversation analysis [20] | Focuses on how participants respond to each other in back-to-forth conversations, and how understanding of each other’s actions is negotiated within conversation. | Constructionist | Focus on minutiae of conversation (e.g. speech delivery) unsuited to large dataset and likely to neglect content of GDG conversations.  Unsuitable for analysis of interview data (would require exploration of interviewer’s speech).  Unsuitable for tracking development of GDG processes over time. | Not useful  (does not offer ways to reduce dataset) | Not useful |
| Thematic analysis [21] | Broad, generic and versatile method for ‘identifying, analysing and reporting themes within data’ ([21], p79). | Compatible with any epistemological position | Identifying themes in subset of data likely to be crucial as a start point for analysis and data reduction. | Useful  (data could be selected for analysis according to whether they fit emergent themes) | Useful  (themed data excerpts could be coded for constructs derived from theory/evidence) |
| Interpretative phenomenological analysis [22] | Attempts to reveal participants’ experiences and the meanings they attach to them, but acknowledges researcher’s interpretative role in this process. | Phenomenological (i.e. assumes that meaning ‘exists’, but only through interpretation of events and experiences) | Might be useful for analysis of individual interview data. Less suited to GDG transcript analysis, which deals with the social interaction and not individuals’ experiences. | Potentially useful  (could aid coding of individual interview data) | Not useful  (emphasis on participants’ experiences may make theory-driven analysis inappropriate) |
| Framework analysis [23] | Involves coding and classifying data into themes in accordance with an analytic framework that is either preconceived or developed iteratively. Resultant accounts of data can be explanatory and/or descriptive. | Compatible with any epistemological position | Useful for organising data into themes. | Useful  (can be used to organise data into themes) | Useful  (allows for theoretical preconceptions, assumptions and hypotheses to be used in analysis) |
